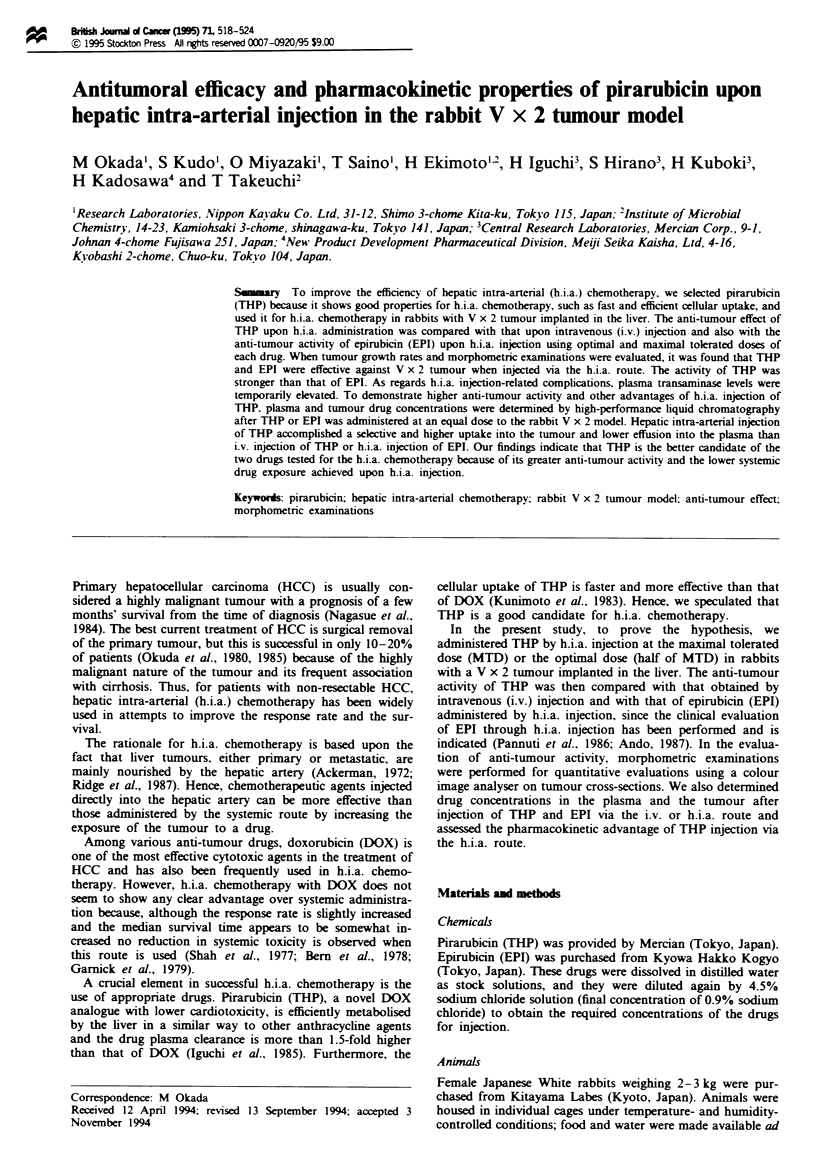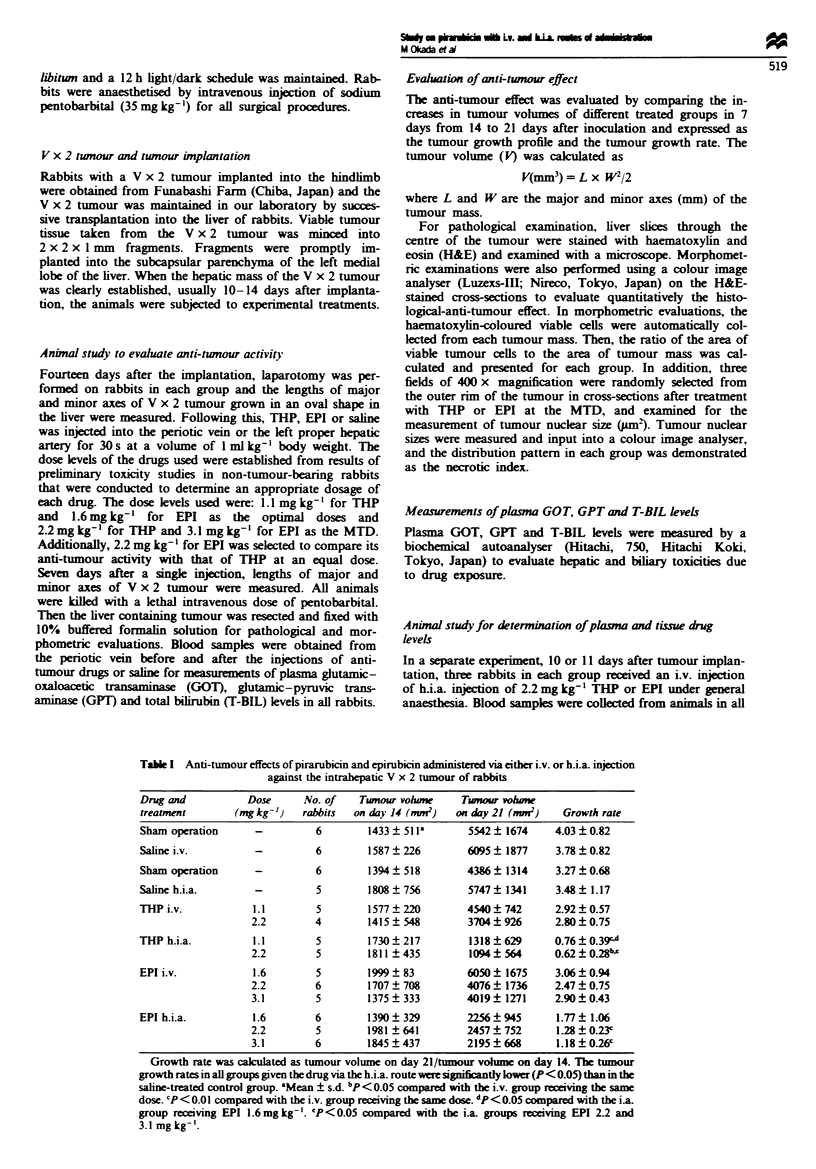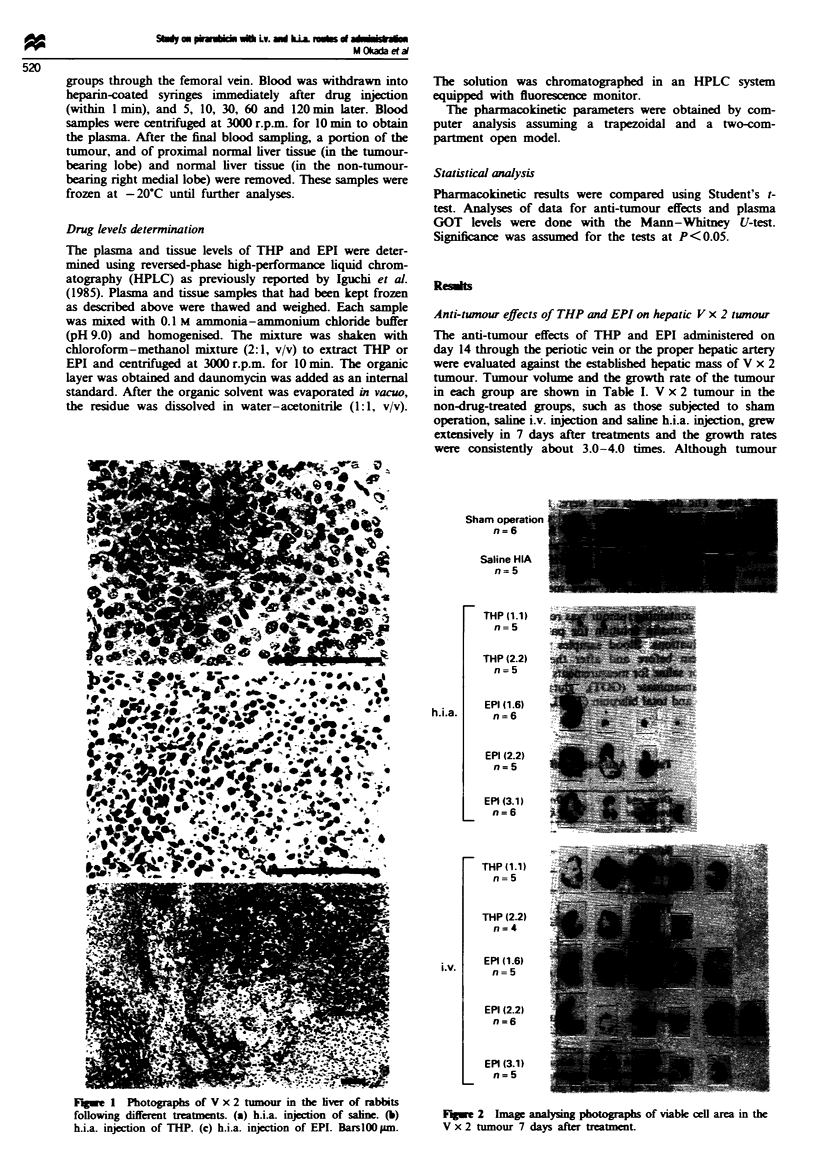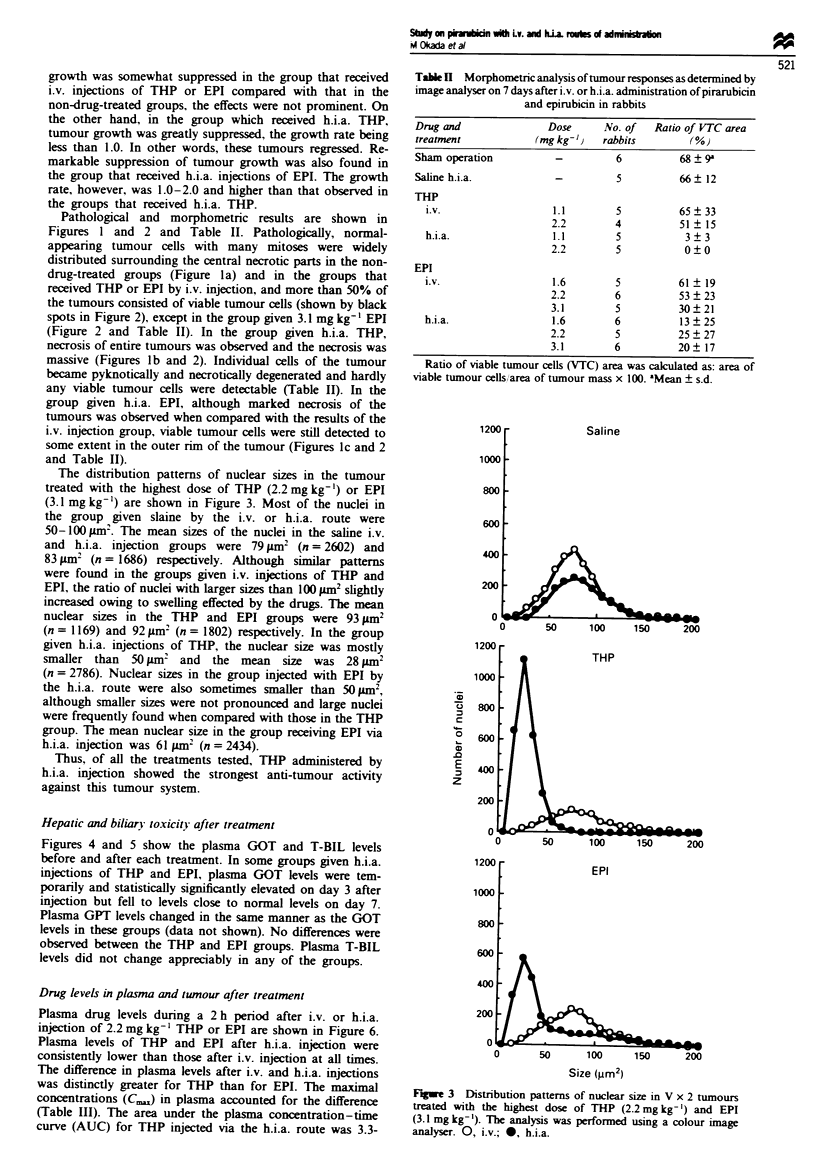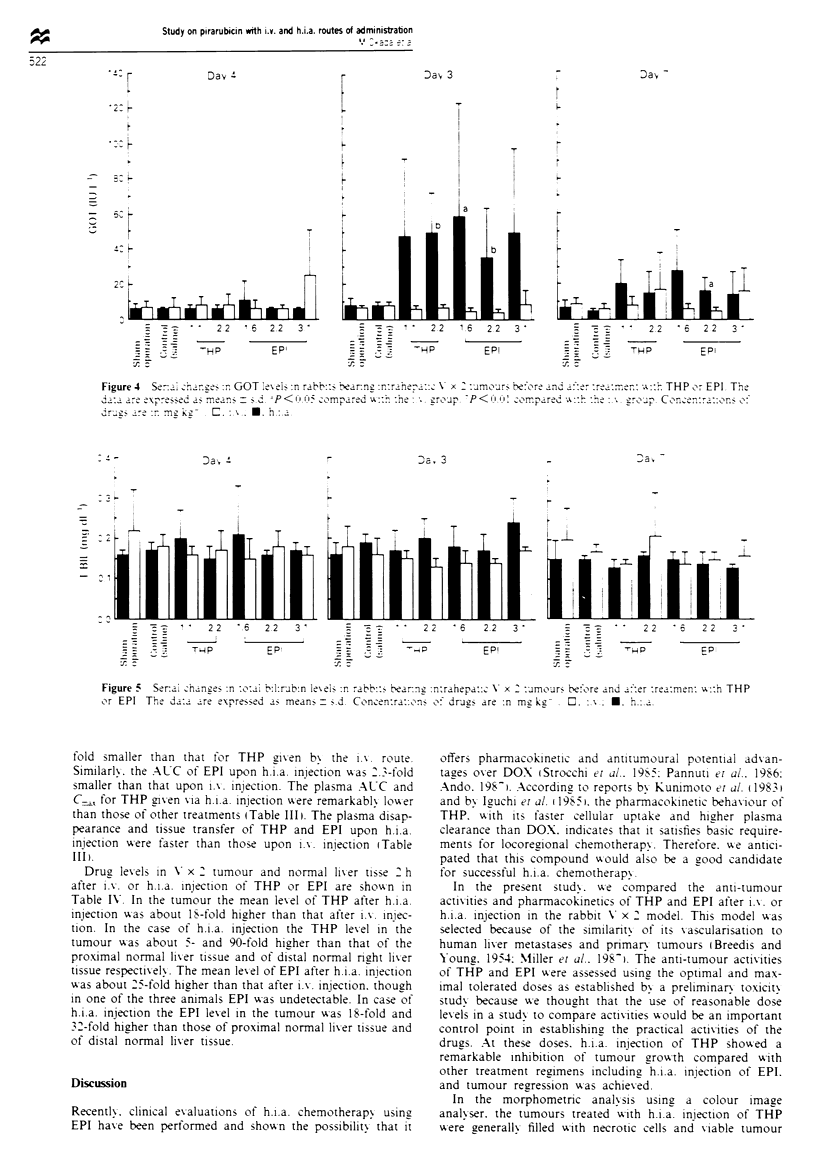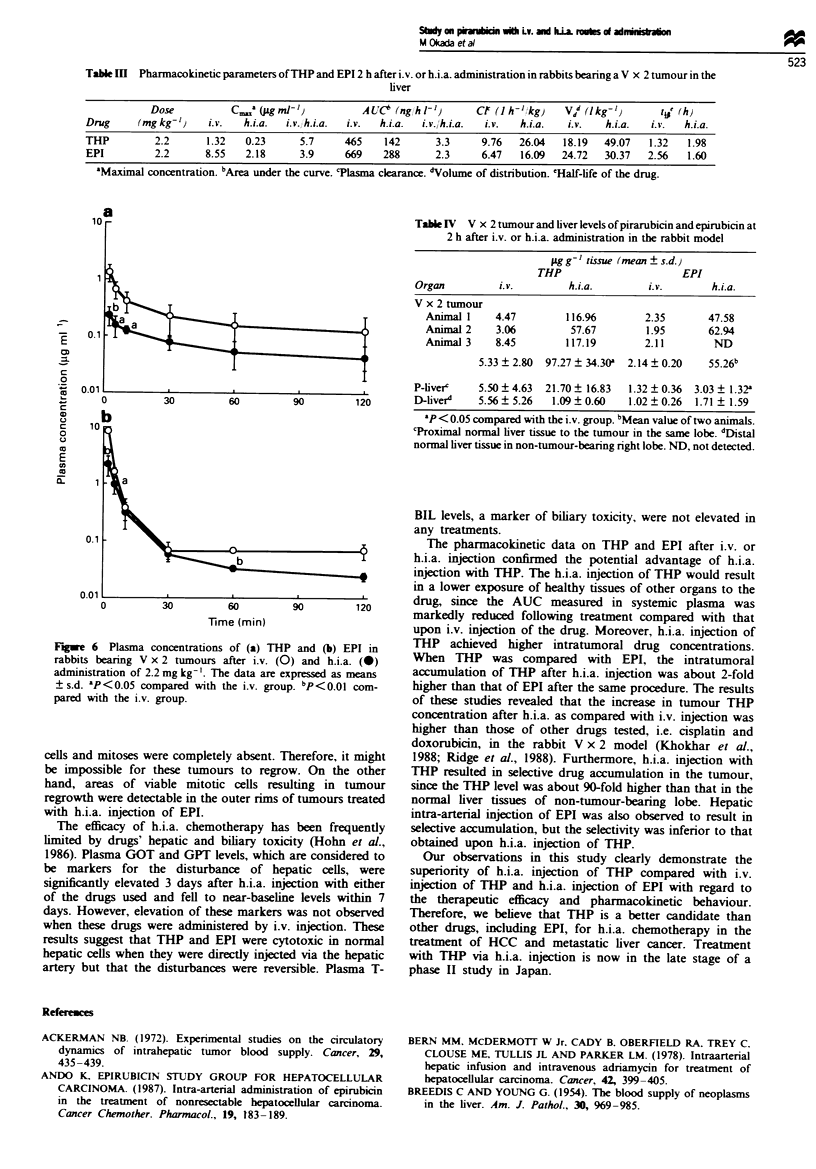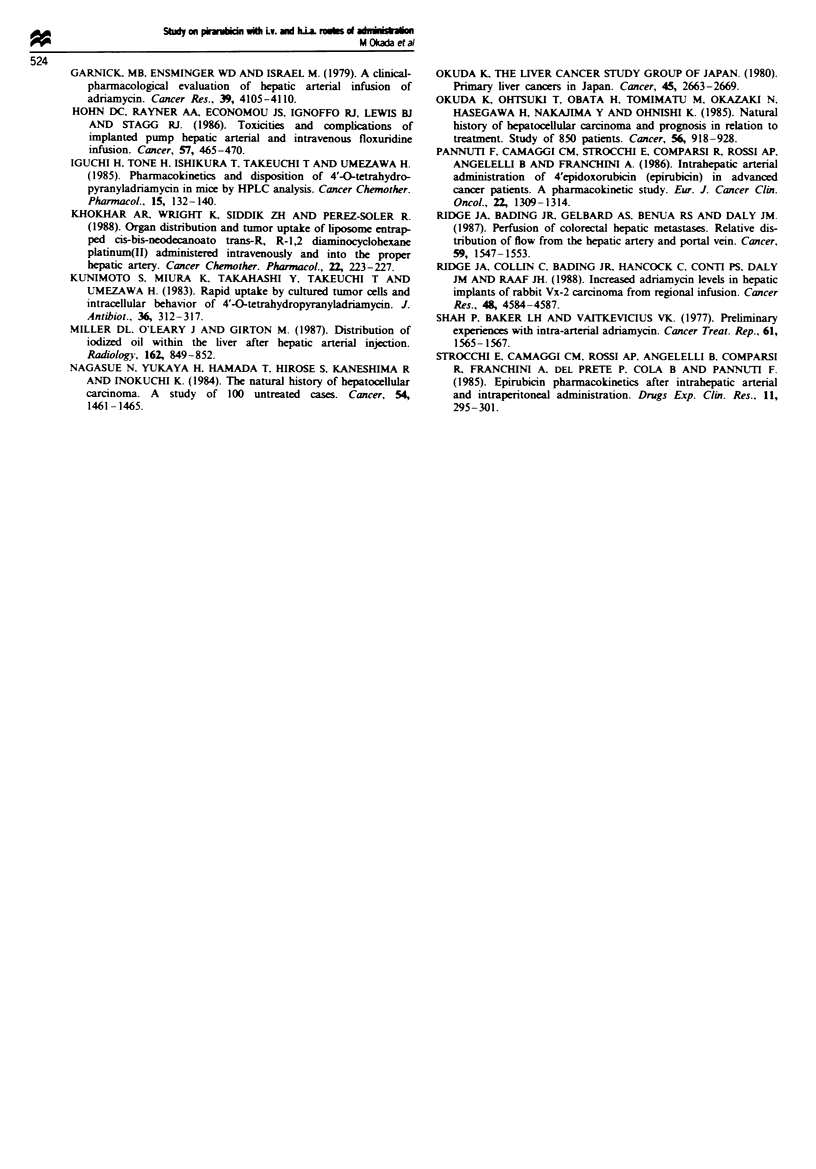# Antitumoral efficacy and pharmacokinetic properties of pirarubicin upon hepatic intra-arterial injection in the rabbit V x 2 tumour model.

**DOI:** 10.1038/bjc.1995.103

**Published:** 1995-03

**Authors:** M. Okada, S. Kudo, O. Miyazaki, T. Saino, H. Ekimoto, H. Iguchi, S. Hirano, H. Kuboki, H. Kadosawa, T. Takeuchi

**Affiliations:** Research Laboratories, Nippon Kayaku Co. Ltd., Tokyo, Japan.

## Abstract

**Images:**


British Journal of Cancer (1995) 71, 518-524

© 1995 Stockton Press All rights reserved 0007-0920/95 $9.00

### Antitumoral efficacy and pharmacokinetic properties of pirarubicin upon hepatic intra-arterial injection in the rabbit V × 2 tumour model

M Okada[1], S Kudo[1], O Miyazaki[1], T Saino[1], H Ekimoto[1,2], H Iguchi[3], S Hirano[3], H Kuboki[3], H Kadosawa[4] and T Takeuchi[2]

[1]*Research Laboratories, Nippon Kayaku Co. Ltd, 31-12, Shimo 3-chome Kita-ku, Tokyo 115, Japan;* [2]*Institute of Microbial Chemistry, 14-23, Kamiohsaki 3-chome, shinagawa-ku, Tokyo 141, Japan;* [3]*Central Research Laboratories, Mercian Corp., 9-1, Johnan 4-chome Fujisawa 251, Japan;* [4]*New Product Development Pharmaceutical Division, Meiji Seika Kaisha, Ltd, 4-16, Kyobashi 2-chome, Chuo-ku, Tokyo 104, Japan.*

**Summary** To improve the efficiency of hepatic intra-arterial (h.i.a.) chemotherapy, we selected pirarubicin (THP) because it shows good properties for h.i.a. chemotherapy, such as fast and efficient cellular uptake, and used it for h.i.a. chemotherapy in rabbits with V x 2 tumour implanted in the liver. The anti-tumour effect of THP upon h.i.a. administration was compared with that upon intravenous (i.v.) injection and also with the anti-tumour activity of epirubicin (EPI) upon h.i.a. injection using optimal and maximal tolerated doses of each drug. When tumour growth rates and morphometric examinations were evaluated, it was found that THP and EPI were effective against V x 2 tumour when injected via the h.i.a. route. The activity of THP was stronger than that of EPI. As regards h.i.a. injection-related complications, plasma transaminase levels were temporarily elevated. To demonstrate higher anti-tumour activity and other advantages of h.i.a. injection of THP, plasma and tumour drug concentrations were determined by high-performance liquid chromatography after THP or EPI was administered at an equal dose to the rabbit V x 2 model. Hepatic intra-arterial injection of THP accomplished a selective and higher uptake into the tumour and lower effusion into the plasma than i.v. injection of THP or h.i.a. injection of EPI. Our findings indicate that THP is the better candidate of the two drugs tested for the h.i.a. chemotherapy because of its greater anti-tumour activity and the lower systemic drug exposure achieved upon h.i.a. injection.

**Keywords**: pirarubicin; hepatic intra-arterial chemotherapy; rabbit V x 2 tumour model; anti-tumour effect; morphometric examinations

Primary hepatocellular carcinoma (HCC) is usually considered a highly malignant tumour with a prognosis of a few months' survival from the time of diagnosis (Nagasue *et al.*, 1984). The best current treatment of HCC is surgical removal of the primary tumour, but this is successful in only 10-20% of patients (Okuda *et al.*, 1980, 1985) because of the highly malignant nature of the tumour and its frequent association with cirrhosis. Thus, for patients with non-resectable HCC, hepatic intra-arterial (h.i.a.) chemotherapy has been widely used in attempts to improve the response rate and the survival.

The rationale for h.i.a. chemotherapy is based upon the fact that liver tumours, either primary or metastatic, are mainly nourished by the hepatic artery (Ackerman, 1972; Ridge *et al.*, 1987). Hence, chemotherapeutic agents injected directly into the hepatic artery can be more effective than those administered by the systemic route by increasing the exposure of the tumour to a drug.

Among various anti-tumour drugs, doxorubicin (DOX) is one of the most effective cytotoxic agents in the treatment of HCC and has also been frequently used in h.i.a. chemotherapy. However, h.i.a. chemotherapy with DOX does not seem to show any clear advantage over systemic administration because, although the response rate is slightly increased and the median survival time appears to be somewhat increased no reduction in systemic toxicity is observed when this route is used (Shah *et al.*, 1977; Bern *et al.*, 1978; Garnick *et al.*, 1979).

A crucial element in successful h.i.a. chemotherapy is the use of appropriate drugs. Pirarubicin (THP), a novel DOX analogue with lower cardiotoxicity, is efficiently metabolised by the liver in a similar way to other anthracycline agents and the drug plasma clearance is more than 1.5-fold higher than that of DOX (Iguchi *et al.*, 1985). Furthermore, the cellular uptake of THP is faster and more effective than that of DOX (Kunimoto *et al.*, 1983). Hence, we speculated that THP is a good candidate for h.i.a. chemotherapy.

In the present study, to prove the hypothesis, we administered THP by h.i.a. injection at the maximal tolerated dose (MTD) or the optimal dose (half of MTD) in rabbits with a V x 2 tumour implanted in the liver. The anti-tumour activity of THP was then compared with that obtained by intravenous (i.v.) injection and with that of epirubicin (EPI) administered by h.i.a. injection, since the clinical evaluation of EPI through h.i.a. injection has been performed and is indicated (Pannuti *et al.*, 1986; Ando, 1987). In the evaluation of anti-tumour activity, morphometric examinations were performed for quantitative evaluations using a colour image analyser on tumour cross-sections. We also determined drug concentrations in the plasma and the tumour after injection of THP and EPI via the i.v. or h.i.a. route and assessed the pharmacokinetic advantage of THP injection via the h.i.a. route.

### Materials and methods

### *Chemicals*

Pirarubicin (THP) was provided by Mercian (Tokyo, Japan). Epirubicin (EPI) was purchased from Kyowa Hakko Kogyo (Tokyo, Japan). These drugs were dissolved in distilled water as stock solutions, and they were diluted again by 4.5% sodium chloride solution (final concentration of 0.9% sodium chloride) to obtain the required concentrations of the drugs for injection.

### *Animals*

Female Japanese White rabbits weighing 2-3 kg were purchased from Kitayama Labes (Kyoto, Japan). Animals were housed in individual cages under temperature- and humidity-controlled conditions; food and water were made available *ad*

Correspondence: M Okada
Received 12 April 1994; revised 13 September 1994; accepted 3 November 1994

*libitum* and a 12 h light/dark schedule was maintained. Rabbits were anaesthetised by intravenous injection of sodium pentobarbital (35 mg $kg^{-1}$) for all surgical procedures.

### *V × 2 tumour and tumour implantation*

Rabbits with a V × 2 tumour implanted into the hindlimb were obtained from Funabashi Farm (Chiba, Japan) and the V × 2 tumour was maintained in our laboratory by successive transplantation into the liver of rabbits. Viable tumour tissue taken from the V × 2 tumour was minced into 2 × 2 × 1 mm fragments. Fragments were promptly implanted into the subcapsular parenchyma of the left medial lobe of the liver. When the hepatic mass of the V × 2 tumour was clearly established, usually 10–14 days after implantation, the animals were subjected to experimental treatments.

### *Animal study to evaluate anti-tumour activity*

Fourteen days after the implantation, laparotomy was performed on rabbits in each group and the lengths of major and minor axes of V × 2 tumour grown in an oval shape in the liver were measured. Following this, THP, EPI or saline was injected into the periotic vein or the left proper hepatic artery for 30 s at a volume of 1 ml $kg^{-1}$ body weight. The dose levels of the drugs used were established from results of preliminary toxicity studies in non-tumour-bearing rabbits that were conducted to determine an appropriate dosage of each drug. The dose levels used were: 1.1 mg $kg^{-1}$ for THP and 1.6 mg $kg^{-1}$ for EPI as the optimal doses and 2.2 mg $kg^{-1}$ for THP and 3.1 mg $kg^{-1}$ for EPI as the MTD. Additionally, 2.2 mg $kg^{-1}$ for EPI was selected to compare its anti-tumour activity with that of THP at an equal dose. Seven days after a single injection, lengths of major and minor axes of V × 2 tumour were measured. All animals were killed with a lethal intravenous dose of pentobarbital. Then the liver containing tumour was resected and fixed with 10% buffered formalin solution for pathological and morphometric evaluations. Blood samples were obtained from the periotic vein before and after the injections of anti-tumour drugs or saline for measurements of plasma glutamic-oxaloacetic transaminase (GOT), glutamic-pyruvic transaminase (GPT) and total bilirubin (T-BIL) levels in all rabbits.

### *Evaluation of anti-tumour effect*

The anti-tumour effect was evaluated by comparing the increases in tumour volumes of different treated groups in 7 days from 14 to 21 days after inoculation and expressed as the tumour growth profile and the tumour growth rate. The tumour volume ($V$) was calculated as

$$V(\text{mm}^3) = L \times W^2/2$$

where $L$ and $W$ are the major and minor axes (mm) of the tumour mass.

For pathological examination, liver slices through the centre of the tumour were stained with haematoxylin and eosin (H&E) and examined with a microscope. Morphometric examinations were also performed using a colour image analyser (Luzexs-III; Nireco, Tokyo, Japan) on the H&E-stained cross-sections to evaluate quantitatively the histological-anti-tumour effect. In morphometric evaluations, the haematoxylin-coloured viable cells were automatically collected from each tumour mass. Then, the ratio of the area of viable tumour cells to the area of tumour mass was calculated and presented for each group. In addition, three fields of 400 × magnification were randomly selected from the outer rim of the tumour in cross-sections after treatment with THP or EPI at the MTD, and examined for the measurement of tumour nuclear size ($\mu m^2$). Tumour nuclear sizes were measured and input into a colour image analyser, and the distribution pattern in each group was demonstrated as the necrotic index.

### *Measurements of plasma GOT, GPT and T-BIL levels*

Plasma GOT, GPT and T-BIL levels were measured by a biochemical autoanalyser (Hitachi, 750, Hitachi Koki, Tokyo, Japan) to evaluate hepatic and biliary toxicities due to drug exposure.

### *Animal study for determination of plasma and tissue drug levels*

In a separate experiment, 10 or 11 days after tumour implantation, three rabbits in each group received an i.v. injection of h.i.a. injection of 2.2 mg $kg^{-1}$ THP or EPI under general anaesthesia. Blood samples were collected from animals in all

**Table I** Anti-tumour effects of pirarubicin and epirubicin administered via either i.v. or h.i.a. injection against the intrahepatic V × 2 tumour of rabbits

| *Drug and treatment* | *Dose (mg $kg^{-1}$)* | *No. of rabbits* | *Tumour volume on day 14 ($mm^3$)* | *Tumour volume on day 21 ($mm^3$)* | *Growth rate* |
|---|---|---|---|---|---|
| Sham operation | – | 6 | 1433 ± 511[a] | 5542 ± 1674 | 4.03 ± 0.82 |
| Saline i.v. | – | 6 | 1587 ± 226 | 6095 ± 1877 | 3.78 ± 0.82 |
| Sham operation | – | 6 | 1394 ± 518 | 4386 ± 1314 | 3.27 ± 0.68 |
| Saline h.i.a. | – | 5 | 1808 ± 756 | 5747 ± 1341 | 3.48 ± 1.17 |
| THP i.v. | 1.1 | 5 | 1577 ± 220 | 4540 ± 742 | 2.92 ± 0.57 |
| | 2.2 | 4 | 1415 ± 548 | 3704 ± 926 | 2.80 ± 0.75 |
| THP h.i.a. | 1.1 | 5 | 1730 ± 217 | 1318 ± 629 | 0.76 ± 0.39[c,d] |
| | 2.2 | 5 | 1811 ± 435 | 1094 ± 564 | 0.62 ± 0.28[b,e] |
| EPI i.v. | 1.6 | 5 | 1999 ± 83 | 6050 ± 1675 | 3.06 ± 0.94 |
| | 2.2 | 6 | 1707 ± 708 | 4076 ± 1736 | 2.47 ± 0.75 |
| | 3.1 | 5 | 1375 ± 333 | 4019 ± 1271 | 2.90 ± 0.43 |
| EPI h.i.a. | 1.6 | 6 | 1390 ± 329 | 2256 ± 945 | 1.77 ± 1.06 |
| | 2.2 | 5 | 1981 ± 641 | 2457 ± 752 | 1.28 ± 0.23[c] |
| | 3.1 | 6 | 1845 ± 437 | 2195 ± 668 | 1.18 ± 0.26[c] |

Growth rate was calculated as tumour volume on day 21/tumour volume on day 14. The tumour growth rates in all groups given the drug via the h.i.a. route were significantly lower ($P < 0.05$) than in the saline-treated control group. [a]Mean ± s.d. [b]$P < 0.05$ compared with the i.v. group receiving the same dose. [c]$P < 0.01$ compared with the i.v. group receiving the same dose. [d]$P < 0.05$ compared with the i.a. group receiving EPI 1.6 mg $kg^{-1}$. [e]$P < 0.05$ compared with the i.a. groups receiving EPI 2.2 and 3.1 mg $kg^{-1}$.

groups through the femoral vein. Blood was withdrawn into heparin-coated syringes immediately after drug injection (within 1 min), and 5, 10, 30, 60 and 120 min later. Blood samples were centrifuged at 3000 r.p.m. for 10 min to obtain the plasma. After the final blood sampling, a portion of the tumour, and of proximal normal liver tissue (in the tumour-bearing lobe) and normal liver tissue (in the non-tumour-bearing right medial lobe) were removed. These samples were frozen at −20°C until further analyses.

*Drug levels determination*

The plasma and tissue levels of THP and EPI were determined using reversed-phase high-performance liquid chromatography (HPLC) as previously reported by Iguchi *et al.* (1985). Plasma and tissue samples that had been kept frozen as described above were thawed and weighed. Each sample was mixed with 0.1 M ammonia–ammonium chloride buffer (pH 9.0) and homogenised. The mixture was shaken with chloroform–methanol mixture (2:1, v/v) to extract THP or EPI and centrifuged at 3000 r.p.m. for 10 min. The organic layer was obtained and daunomycin was added as an internal standard. After the organic solvent was evaporated *in vacuo*, the residue was dissolved in water–acetonitrile (1:1, v/v). The solution was chromatographed in an HPLC system equipped with fluorescence monitor.

The pharmacokinetic parameters were obtained by computer analysis assuming a trapezoidal and a two-compartment open model.

*Statistical analysis*

Pharmacokinetic results were compared using Student's *t*-test. Analyses of data for anti-tumour effects and plasma GOT levels were done with the Mann–Whitney *U*-test. Significance was assumed for the tests at $P<0.05$.

### Results

*Anti-tumour effects of THP and EPI on hepatic V × 2 tumour*

The anti-tumour effects of THP and EPI administered on day 14 through the periotic vein or the proper hepatic artery were evaluated against the established hepatic mass of V × 2 tumour. Tumour volume and the growth rate of the tumour in each group are shown in Table I. V × 2 tumour in the non-drug-treated groups, such as those subjected to sham operation, saline i.v. injection and saline h.i.a. injection, grew extensively in 7 days after treatments and the growth rates were consistently about 3.0–4.0 times. Although tumour

**Figure 1** Photographs of V × 2 tumour in the liver of rabbits following different treatments. (**a**) h.i.a. injection of saline. (**b**) h.i.a. injection of THP. (**c**) h.i.a. injection of EPI. Bars100 μm.

**Figure 2** Image analysing photographs of viable cell area in the V × 2 tumour 7 days after treatment.

growth was somewhat suppressed in the group that received i.v. injections of THP or EPI compared with that in the non-drug-treated groups, the effects were not prominent. On the other hand, in the group which received h.i.a. THP, tumour growth was greatly suppressed, the growth rate being less than 1.0. In other words, these tumours regressed. Remarkable suppression of tumour growth was also found in the group that received h.i.a. injections of EPI. The growth rate, however, was 1.0–2.0 and higher than that observed in the groups that received h.i.a. THP.

Pathological and morphometric results are shown in Figures 1 and 2 and Table II. Pathologically, normal-appearing tumour cells with many mitoses were widely distributed surrounding the central necrotic parts in the non-drug-treated groups (Figure 1a) and in the groups that received THP or EPI by i.v. injection, and more than 50% of the tumours consisted of viable tumour cells (shown by black spots in Figure 2), except in the group given 3.1 mg $kg^{-1}$ EPI (Figure 2 and Table II). In the group given h.i.a. THP, necrosis of entire tumours was observed and the necrosis was massive (Figures 1b and 2). Individual cells of the tumour became pyknotically and necrotically degenerated and hardly any viable tumour cells were detectable (Table II). In the group given h.i.a. EPI, although marked necrosis of the tumours was observed when compared with the results of the i.v. injection group, viable tumour cells were still detected to some extent in the outer rim of the tumour (Figures 1c and 2 and Table II).

The distribution patterns of nuclear sizes in the tumour treated with the highest dose of THP (2.2 mg $kg^{-1}$) or EPI (3.1 mg $kg^{-1}$) are shown in Figure 3. Most of the nuclei in the group given slaine by the i.v. or h.i.a. route were 50–100 $\mu m^2$. The mean sizes of the nuclei in the saline i.v. and h.i.a. injection groups were 79 $\mu m^2$ ($n = 2602$) and 83 $\mu m^2$ ($n = 1686$) respectively. Although similar patterns were found in the groups given i.v. injections of THP and EPI, the ratio of nuclei with larger sizes than 100 $\mu m^2$ slightly increased owing to swelling effected by the drugs. The mean nuclear sizes in the THP and EPI groups were 93 $\mu m^2$ ($n = 1169$) and 92 $\mu m^2$ ($n = 1802$) respectively. In the group given h.i.a. injections of THP, the nuclear size was mostly smaller than 50 $\mu m^2$ and the mean size was 28 $\mu m^2$ ($n = 2786$). Nuclear sizes in the group injected with EPI by the h.i.a. route were also sometimes smaller than 50 $\mu m^2$, although smaller sizes were not pronounced and large nuclei were frequently found when compared with those in the THP group. The mean nuclear size in the group receiving EPI via h.i.a. injection was 61 $\mu m^2$ ($n = 2434$).

Thus, of all the treatments tested, THP administered by h.i.a. injection showed the strongest anti-tumour activity against this tumour system.

### *Hepatic and biliary toxicity after treatment*

Figures 4 and 5 show the plasma GOT and T-BIL levels before and after each treatment. In some groups given h.i.a. injections of THP and EPI, plasma GOT levels were temporarily and statistically significantly elevated on day 3 after injection but fell to levels close to normal levels on day 7. Plasma GPT levels changed in the same manner as the GOT levels in these groups (data not shown). No differences were observed between the THP and EPI groups. Plasma T-BIL levels did not change appreciably in any of the groups.

### *Drug levels in plasma and tumour after treatment*

Plasma drug levels during a 2 h period after i.v. or h.i.a. injection of 2.2 mg $kg^{-1}$ THP or EPI are shown in Figure 6. Plasma levels of THP and EPI after h.i.a. injection were consistently lower than those after i.v. injection at all times. The difference in plasma levels after i.v. and h.i.a. injections was distinctly greater for THP than for EPI. The maximal concentrations ($C_{max}$) in plasma accounted for the difference (Table III). The area under the plasma concentration–time curve (AUC) for THP injected via the h.i.a. route was 3.3-

**Table II** Morphometric analysis of tumour responses as determined by image analyser on 7 days after i.v. or h.i.a. administration of pirarubicin and epirubicin in rabbits

| *Drug and treatment* | *Dose (mg $kg^{-1}$)* | *No. of rabbits* | *Ratio of VTC area (%)* |
|---|---|---|---|
| Sham operation | – | 6 | 68 ± 9[a] |
| Saline h.i.a. | – | 5 | 66 ± 12 |
| THP | | | |
| i.v. | 1.1 | 5 | 65 ± 33 |
| | 2.2 | 4 | 51 ± 15 |
| h.i.a. | 1.1 | 5 | 3 ± 3 |
| | 2.2 | 5 | 0 ± 0 |
| EPI | | | |
| i.v. | 1.6 | 5 | 61 ± 19 |
| | 2.2 | 6 | 53 ± 23 |
| | 3.1 | 5 | 30 ± 21 |
| h.i.a. | 1.6 | 6 | 13 ± 25 |
| | 2.2 | 5 | 25 ± 27 |
| | 3.1 | 6 | 20 ± 17 |

Ratio of viable tumour cells (VTC) area was calculated as: area of viable tumour cells/area of tumour mass × 100. [a]Mean ± s.d.

**Figure 3** Distribution patterns of nuclear size in V × 2 tumours treated with the highest dose of THP (2.2 mg $kg^{-1}$) and EPI (3.1 mg $kg^{-1}$). The analysis was performed using a colour image analyser. ○, i.v.; ●, h.i.a.

fold smaller than that for THP given by the i.v. route. Similarly, the AUC of EPI upon h.i.a. injection was 2.3-fold smaller than that upon i.v. injection. The plasma AUC and $C_{max}$ for THP given via h.i.a. injection were remarkably lower than those of other treatments (Table III). The plasma disappearance and tissue transfer of THP and EPI upon h.i.a. injection were faster than those upon i.v. injection (Table III).

Drug levels in VX 2 tumour and normal liver tisse 2 h after i.v. or h.i.a. injection of THP or EPI are shown in Table IV. In the tumour the mean level of THP after h.i.a. injection was about 18-fold higher than that after i.v. injection. In the case of h.i.a. injection the THP level in the tumour was about 5- and 90-fold higher than that of the proximal normal liver tissue and of distal normal right liver tissue respectively. The mean level of EPI after h.i.a. injection was about 25-fold higher than that after i.v. injection, though in one of the three animals EPI was undetectable. In case of h.i.a. injection the EPI level in the tumour was 18-fold and 32-fold higher than those of proximal normal liver tissue and of distal normal liver tissue.

### Discussion

Recently, clinical evaluations of h.i.a. chemotherapy using EPI have been performed and shown the possibility that it offers pharmacokinetic and antitumoural potential advantages over DOX (Strocchi *et al.*, 1985; Pannuti *et al.*, 1986; Ando, 1987). According to reports by Kunimoto *et al.* (1983) and by Iguchi *et al.* (1985), the pharmacokinetic behaviour of THP, with its faster cellular uptake and higher plasma clearance than DOX, indicates that it satisfies basic requirements for locoregional chemotherapy. Therefore, we anticipated that this compound would also be a good candidate for successful h.i.a. chemotherapy.

In the present study, we compared the anti-tumour activities and pharmacokinetics of THP and EPI after i.v. or h.i.a. injection in the rabbit VX 2 model. This model was selected because of the similarity of its vascularisation to human liver metastases and primary tumours (Breedis and Young, 1954; Miller *et al.*, 1987). The anti-tumour activities of THP and EPI were assessed using the optimal and maximal tolerated doses as established by a preliminary toxicity study because we thought that the use of reasonable dose levels in a study to compare activities would be an important control point in establishing the practical activities of the drugs. At these doses, h.i.a. injection of THP showed a remarkable inhibition of tumour growth compared with other treatment regimens including h.i.a. injection of EPI, and tumour regression was achieved.

In the morphometric analysis using a colour image analyser, the tumours treated with h.i.a. injection of THP were generally filled with necrotic cells and viable tumour

**Table III** Pharmacokinetic parameters of THP and EPI 2 h after i.v. or h.i.a. administration in rabbits bearing a V × 2 tumour in the liver

| Drug | Dose ($mg\ kg^{-1}$) | $C_{max}$[a] ($\mu g\ ml^{-1}$) i.v. | $C_{max}$[a] h.i.a. | $C_{max}$[a] i.v./h.i.a. | AUC[b] ($ng\ h\ l^{-1}$) i.v. | AUC[b] h.i.a. | AUC[b] i.v./h.i.a. | Cl[c] ($l\ h^{-1}/kg$) i.v. | Cl[c] h.i.a. | $V_d$[d] ($l\ kg^{-1}$) i.v. | $V_d$[d] h.i.a. | $t_{1/2\beta}$[e] (h) i.v. | $t_{1/2\beta}$[e] h.i.a. |
|---|---|---|---|---|---|---|---|---|---|---|---|---|---|
| THP | 2.2 | 1.32 | 0.23 | 5.7 | 465 | 142 | 3.3 | 9.76 | 26.04 | 18.19 | 49.07 | 1.32 | 1.98 |
| EPI | 2.2 | 8.55 | 2.18 | 3.9 | 669 | 288 | 2.3 | 6.47 | 16.09 | 24.72 | 30.37 | 2.56 | 1.60 |

[a]Maximal concentration. [b]Area under the curve. [c]Plasma clearance. [d]Volume of distribution. [e]Half-life of the drug.

**Figure 6** Plasma concentrations of (**a**) THP and (**b**) EPI in rabbits bearing V × 2 tumours after i.v. (○) and h.i.a. (●) administration of 2.2 mg $kg^{-1}$. The data are expressed as means ± s.d. [a]$P < 0.05$ compared with the i.v. group. [b]$P < 0.01$ compared with the i.v. group.

**Table IV** V × 2 tumour and liver levels of pirarubicin and epirubicin at 2 h after i.v. or h.i.a. administration in the rabbit model

| Organ | THP i.v. ($\mu g\ g^{-1}$ tissue (mean ± s.d.)) | THP h.i.a. | EPI i.v. | EPI h.i.a. |
|---|---|---|---|---|
| V × 2 tumour | | | | |
| Animal 1 | 4.47 | 116.96 | 2.35 | 47.58 |
| Animal 2 | 3.06 | 57.67 | 1.95 | 62.94 |
| Animal 3 | 8.45 | 117.19 | 2.11 | ND |
| | 5.33 ± 2.80 | 97.27 ± 34.30[a] | 2.14 ± 0.20 | 55.26[b] |
| P-liver[c] | 5.50 ± 4.63 | 21.70 ± 16.83 | 1.32 ± 0.36 | 3.03 ± 1.32[a] |
| D-liver[d] | 5.56 ± 5.26 | 1.09 ± 0.60 | 1.02 ± 0.26 | 1.71 ± 1.59 |

[a]$P < 0.05$ compared with the i.v. group. [b]Mean value of two animals. [c]Proximal normal liver tissue to the tumour in the same lobe. [d]Distal normal liver tissue in non-tumour-bearing right lobe. ND, not detected.

cells and mitoses were completely absent. Therefore, it might be impossible for these tumours to regrow. On the other hand, areas of viable mitotic cells resulting in tumour regrowth were detectable in the outer rims of tumours treated with h.i.a. injection of EPI.

The efficacy of h.i.a. chemotherapy has been frequently limited by drugs' hepatic and biliary toxicity (Hohn *et al.*, 1986). Plasma GOT and GPT levels, which are considered to be markers for the disturbance of hepatic cells, were significantly elevated 3 days after h.i.a. injection with either of the drugs used and fell to near-baseline levels within 7 days. However, elevation of these markers was not observed when these drugs were administered by i.v. injection. These results suggest that THP and EPI were cytotoxic in normal hepatic cells when they were directly injected via the hepatic artery but that the disturbances were reversible. Plasma T-BIL levels, a marker of biliary toxicity, were not elevated in any treatments.

The pharmacokinetic data on THP and EPI after i.v. or h.i.a. injection confirmed the potential advantage of h.i.a. injection with THP. The h.i.a. injection of THP would result in a lower exposure of healthy tissues of other organs to the drug, since the AUC measured in systemic plasma was markedly reduced following treatment compared with that upon i.v. injection of the drug. Moreover, h.i.a. injection of THP achieved higher intratumoral drug concentrations. When THP was compared with EPI, the intratumoral accumulation of THP after h.i.a. injection was about 2-fold higher than that of EPI after the same procedure. The results of these studies revealed that the increase in tumour THP concentration after h.i.a. as compared with i.v. injection was higher than those of other drugs tested, i.e. cisplatin and doxorubicin, in the rabbit V × 2 model (Khokhar *et al.*, 1988; Ridge *et al.*, 1988). Furthermore, h.i.a. injection with THP resulted in selective drug accumulation in the tumour, since the THP level was about 90-fold higher than that in the normal liver tissues of non-tumour-bearing lobe. Hepatic intra-arterial injection of EPI was also observed to result in selective accumulation, but the selectivity was inferior to that obtained upon h.i.a. injection of THP.

Our observations in this study clearly demonstrate the superiority of h.i.a. injection of THP compared with i.v. injection of THP and h.i.a. injection of EPI with regard to the therapeutic efficacy and pharmacokinetic behaviour. Therefore, we believe that THP is a better candidate than other drugs, including EPI, for h.i.a. chemotherapy in the treatment of HCC and metastatic liver cancer. Treatment with THP via h.i.a. injection is now in the late stage of a phase II study in Japan.

### References

ACKERMAN NB. (1972). Experimental studies on the circulatory dynamics of intrahepatic tumor blood supply. *Cancer*, **29**, 435–439.

ANDO K, EPIRUBICIN STUDY GROUP FOR HEPATOCELLULAR CARCINOMA. (1987). Intra-arterial administration of epirubicin in the treatment of nonresectable hepatocellular carcinoma. *Cancer Chemother. Pharmacol.*, **19**, 183–189.

BERN MM, McDERMOTT W Jr, CADY B, OBERFIELD RA, TREY C, CLOUSE ME, TULLIS JL AND PARKER LM. (1978). Intraarterial hepatic infusion and intravenous adriamycin for treatment of hepatocellular carcinoma. *Cancer*, **42**, 399–405.

BREEDIS C AND YOUNG G. (1954). The blood supply of neoplasms in the liver. *Am. J. Pathol.*, **30**, 969–985.

GARNICK MB, ENSMINGER WD AND ISRAEL M. (1979). A clinical-pharmacological evaluation of hepatic arterial infusion of adriamycin. *Cancer Res.*, **39**, 4105-4110.

HOHN DC, RAYNER AA, ECONOMOU JS, IGNOFFO RJ, LEWIS BJ AND STAGG RJ. (1986). Toxicities and complications of implanted pump hepatic arterial and intravenous floxuridine infusion. *Cancer*, **57**, 465-470.

IGUCHI H, TONE H, ISHIKURA T, TAKEUCHI T AND UMEZAWA H. (1985). Pharmacokinetics and disposition of 4'-O-tetrahydropyranyladriamycin in mice by HPLC analysis. *Cancer Chemother. Pharmacol.*, **15**, 132-140.

KHOKHAR AR, WRIGHT K, SIDDIK ZH AND PEREZ-SOLER R. (1988). Organ distribution and tumor uptake of liposome entrapped cis-bis-neodecanoato trans-R, R-1,2 diaminocyclohexane platinum(II) administered intravenously and into the proper hepatic artery. *Cancer Chemother. Pharmacol.*, **22**, 223-227.

KUNIMOTO S, MIURA K, TAKAHASHI Y, TAKEUCHI T AND UMEZAWA H. (1983). Rapid uptake by cultured tumor cells and intracellular behavior of 4'-O-tetrahydropyranyladriamycin. *J. Antibiot.*, **36**, 312-317.

MILLER DL, O'LEARY J AND GIRTON M. (1987). Distribution of iodized oil within the liver after hepatic arterial injection. *Radiology*, **162**, 849-852.

NAGASUE N, YUKAYA H, HAMADA T, HIROSE S, KANESHIMA R AND INOKUCHI K. (1984). The natural history of hepatocellular carcinoma. A study of 100 untreated cases. *Cancer*, **54**, 1461-1465.

OKUDA K, THE LIVER CANCER STUDY GROUP OF JAPAN. (1980). Primary liver cancers in Japan. *Cancer*, **45**, 2663-2669.

OKUDA K, OHTSUKI T, OBATA H, TOMIMATU M, OKAZAKI N, HASEGAWA H, NAKAJIMA Y AND OHNISHI K. (1985). Natural history of hepatocellular carcinoma and prognosis in relation to treatment. Study of 850 patients. *Cancer*, **56**, 918-928.

PANNUTI F, CAMAGGI CM, STROCCHI E, COMPARSI R, ROSSI AP, ANGELELLI B AND FRANCHINI A. (1986). Intrahepatic arterial administration of 4'epidoxorubicin (epirubicin) in advanced cancer patients. A pharmacokinetic study. *Eur. J. Cancer Clin. Oncol.*, **22**, 1309-1314.

RIDGE JA, BADING JR, GELBARD AS, BENUA RS AND DALY JM. (1987). Perfusion of colorectal hepatic metastases. Relative distribution of flow from the hepatic artery and portal vein. *Cancer*, **59**, 1547-1553.

RIDGE JA, COLLIN C, BADING JR, HANCOCK C, CONTI PS, DALY JM AND RAAF JH. (1988). Increased adriamycin levels in hepatic implants of rabbit Vx-2 carcinoma from regional infusion. *Cancer Res.*, **48**, 4584-4587.

SHAH P, BAKER LH AND VAITKEVICIUS VK. (1977). Preliminary experiences with intra-arterial adriamycin. *Cancer Treat. Rep.*, **61**, 1565-1567.

STROCCHI E, CAMAGGI CM, ROSSI AP, ANGELELLI B, COMPARSI R, FRANCHINI A, DEL PRETE P, COLA B AND PANNUTI F. (1985). Epirubicin pharmacokinetics after intrahepatic arterial and intraperitoneal administration. *Drugs Exp. Clin. Res.*, **11**, 295-301.